# Multielectrode Radiofrequency Balloon Catheter for Paroxysmal Atrial Fibrillation: Results From the Global, Multicenter, STELLAR Study

**DOI:** 10.1111/jce.16524

**Published:** 2024-12-16

**Authors:** Sandeep K. Goyal, Carlo Pappone, Massimo Grimaldi, Sung W. Lee, Stavros Mountantonakis, J. Brian DeVille, Venkata S. Sagi, Chen‐Yang Jiang, Haseeb Jafri, Alan P. Wimmer, Li‐Qun Wu, Srinivas Dukkipati, Haroon Rashid, Hugh Calkins, Moussa Mansour, Javier Roman‐Gonzalez, Andrea Natale, Giuseppe Ciconte, Arash Aryana, STELLAR investigators

**Affiliations:** ^1^ Division of Cardiac Electrophysiology, Piedmont Heart Institute Atlanta Georgia USA; ^2^ Department of Arrhythmology IRCCS Policlinico San Donato, San Donato Milanese Milan Italy; ^3^ Department of Arrhythmology Vita‐Salute San Raffaele University Milan Italy; ^4^ Department of Cardiology General Regional Hospital F. Miulli Bari Italy; ^5^ Division of Cardiology MedStar Southern Maryland Hospital Center Clinton Maryland USA; ^6^ Department of Cardiac Electrophysiology Northwell Health‐Lenox Hill Hospital New York City New York USA; ^7^ Division of Cardiology The Heart Hospital Baylor Plano Texas USA; ^8^ Baptist Heart Specialists Baptist Medical Center Jacksonville Florida USA; ^9^ Department of Cardiology Sir Run Run Shaw Hospital, College of Medicine, Zhejiang University Hangzhou China; ^10^ Kettering Physician Network Cardiac Electrophysiology Kettering Ohio USA; ^11^ Division of Cardiology Saint Luke's Mid America Heart Institute Kansas City Missouri USA; ^12^ Division of Cardiology University of Missouri Kansas City School of Medicine Kansas City Missouri USA; ^13^ Department of Cardiology Ruijin Hospital, Shanghai Jiao Tong University School of Medicine Shanghai China; ^14^ Department of Cardiology Icahn School of Medicine at Mount Sinai New York City New York USA; ^15^ Virginia Heart, Heart Rhythm Center Falls Church Virginia USA; ^16^ Division of Cardiology, Department of Medicine Johns Hopkins University School of Medicine Baltimore Maryland USA; ^17^ Cardiac Arrhythmia Center, Massachusetts General Hospital, Harvard Medical School Boston Massachusetts USA; ^18^ South Texas Cardiovascular Consultants, Texan Medical Center San Antonio Texas USA; ^19^ Texas Cardiac Arrhythmia Institute, St. David's Medical Center Austin Texas USA; ^20^ Division of Cardiology, Department of Biomedicine and Prevention University of Tor Vergata Rome Italy; ^21^ Metro Health Medical Center, Case Western Reserve University School of Medicine Cleveland Ohio USA; ^22^ Interventional Electrophysiology, Scripps Clinic San Diego California USA; ^23^ Mercy General Hospital and Dignity Health Heart and Vascular Institute Sacramento California USA

**Keywords:** drug‐refractory, paroxysmal atrial fibrillation, radiofrequency balloon catheter

## Abstract

**Introduction:**

The safety and efficacy of paroxysmal atrial fibrillation (PAF) ablation with the HELIOSTAR multielectrode radiofrequency (RF) balloon catheter have been demonstrated in European studies; data from elsewhere are lacking. This prospective, multicenter study conducted in the United States, Italy, and China investigated the safety and efficacy of pulmonary vein isolation (PVI) using HELIOSTAR in drug‐refractory symptomatic PAF.

**Methods:**

The primary effectiveness endpoint (PEE) was 12‐month freedom from documented atrial fibrillation/atrial flutter/atrial tachycardia plus freedom from acute procedural failure, nonstudy catheter failure, repeat ablation failure, direct current cardioversion (DCCV), and Class I/III antiarrhythmic drug (AAD) failure. The primary safety endpoint was the occurrence of early‐onset primary adverse events (PAEs). Cerebral magnetic resonance imaging (MRI) and cardiac computed tomography were performed in a patient subset to assess silent cerebral lesions (SCLs) and severe pulmonary vein (PV) stenosis, respectively.

**Results:**

Across 36 centers, 257 eligible subjects in the main phase had the study catheter inserted. Acute PVI was achieved in all subjects, with the majority (94.1%) using the balloon catheter only. In 67.7% and 92.2% of subjects, respectively, PEE and freedom from repeat ablation were met; clinical success rate was 77.7%. The PAE rate was 5.1%. One of 15 (6.7%) subjects with MRI showed a new SCL at 1 month postablation, which resolved at 3 months. Clinically meaningful improvements in Atrial Fibrillation Effect on QualiTy‐of‐life scores were seen at 3 months and were sustained to 12 months postablation, and accompanied with reduction of Class I/III AAD use and DCCV.

**Conclusion:**

STELLAR confirmed the safety and efficacy of the HELIOSTAR catheter for PVI, with clinically meaningful improvements in quality of life in patients with drug‐refractory symptomatic PAF. Most PVIs were achieved without focal touch‐up, and > 90% of patients were free from repeat ablation at 12 months.

**Trial Registration:**

ClinicalTrials.gov Identifier: NCT03683030.

## Graphical abstract

**Central Illustration.** AAD, antiarrhythmic drug; AFEQT, Atrial Fibrillation Effect on Quality‐of‐Life; CHA_2_DS_2_‐VASc, congestive heart failure, hypertension, age ≥75 years (doubled), diabetes mellitus, stroke/transient ischemic attack/thromboembolism (doubled), vascular disease, age 65–74 years, sex category; LA, left atrial; LVEF, left ventricular ejection fraction; PAF, paroxysmal atrial fibrillation. *An increase of ≥ 5 points is considered a clinically meaningful improvement. Image is courtesy of © Biosense Webster, Inc., part of Johnson & Johnson MedTech. All rights reserved.

**Abbreviations**
AAD: antiarrhythmic drug
AF: atrial fibrillation
AFEQT: atrial fibrillation effect on quality‐of‐life
AFL: atrial flutter
AT: atrial tachycardia
CHA_2_DS_2_‐VASc: congestive heart failure, hypertension, age ≥ 75 years (doubled), diabetes mellitus, stroke/transient ischemic attack/thromboembolism (doubled), vascular disease, age 65–74 years, sex category
CT: computed tomography
CTI: cavotricuspid isthmus
DCCV: direct current cardioversion
ECG: electrocardiogram
FLAIR: fluid‐attenuated inversion recovery
ICE: intracardiac echocardiography
LA: left atrium
LIPV: left inferior pulmonary vein
LPV: left pulmonary vein
LSPV: left superior pulmonary vein
mITT: modified intent‐to‐treat
MRA: magnetic resonance angiogram assessment
MRI: magnetic resonance imaging
NAE: neurologic assessment evaluable
NIHSS: National Institutes of Health Stroke Scale
NYHA: New York Heart Association
PAE: primary adverse event
PAF: paroxysmal atrial fibrillation
PBP: point‐by‐point
PEE: primary effectiveness endpoint
PP: per‐protocol
PV: pulmonary vein
PVI: pulmonary vein isolation
QOL: quality of life
RF: radiofrequency
RIPV: right inferior pulmonary vein
RMPV: right middle pulmonary vein
RSPV: right superior pulmonary vein
SCL: silent cerebral lesion
SD: standard deviation
TTM: transtelephonic monitoring

## 1 Introduction

Point‐by‐point (PBP) cardiac ablation with radiofrequency (RF) catheters has been used successfully in the treatment of paroxysmal atrial fibrillation (PAF). However, it is a technically complex procedure and outcomes may be highly dependent on individual operators’ learning curves, except where a standardized approach to ablation is employed, such as with index‐guided ablation [1, 2]. The development of single‐shot balloon catheters has provided an alternative approach that aims to reduce the technical complexity while improving procedural efficiency [3, 4].

The HELIOSTAR balloon catheter (Biosense Webster, Inc., Irvine, CA, USA) is a 28‐mm diameter compliant multielectrode RF balloon catheter that can conform and be maneuvered to varied pulmonary vein (PV) anatomy for optimal catheter‐tissue contact [5, 6]. The catheter can perform circumferential or segmental ablation with 10 irrigated, flexible gold electrodes, each of which is capable of independently delivering varying levels of RF energy by adjusting for different thicknesses of target tissues. The RADIANCE and SHINE studies demonstrated the safe and effective use of the compliant multielectrode RF balloon catheter in conjunction with the LASSOSTAR (LS) circular diagnostic catheter for electrophysiologic mapping and RF ablation in the treatment of PAF through pulmonary vein isolation (PVI), with strong procedural efficiency, and promising 1‐year arrhythmia‐free and repeat ablation rates [5, 6, 7]. Recent real‐world experience showed the possibility of reaching a high rate of single‐shot isolation (95.8%) while maintaining a low complication rate (3.3%) when following an optimized workflow that takes into consideration preablation electrode impedance and temperature [8]. Additionally, wide‐area antral PVI with concomitant posterior wall isolation, beyond the ostial level, can be achieved safely and effectively utilizing the segmental approach [9]. Another study demonstrated the safety and effectiveness of a circumferential (i.e., nonsegmental) ablation workflow [10].

The above studies were conducted in European countries and populations. Here, we report the 1‐year results of the STELLAR study, which investigated the safety and effectiveness of the compliant multielectrode RF balloon catheter, in conjunction with the LS circular diagnostic catheter, for the treatment of patients with drug‐refractory symptomatic PAF across centers in other parts of the world (Central Illustration 1).

**CENTRAL ILLUSTRATION 1:**
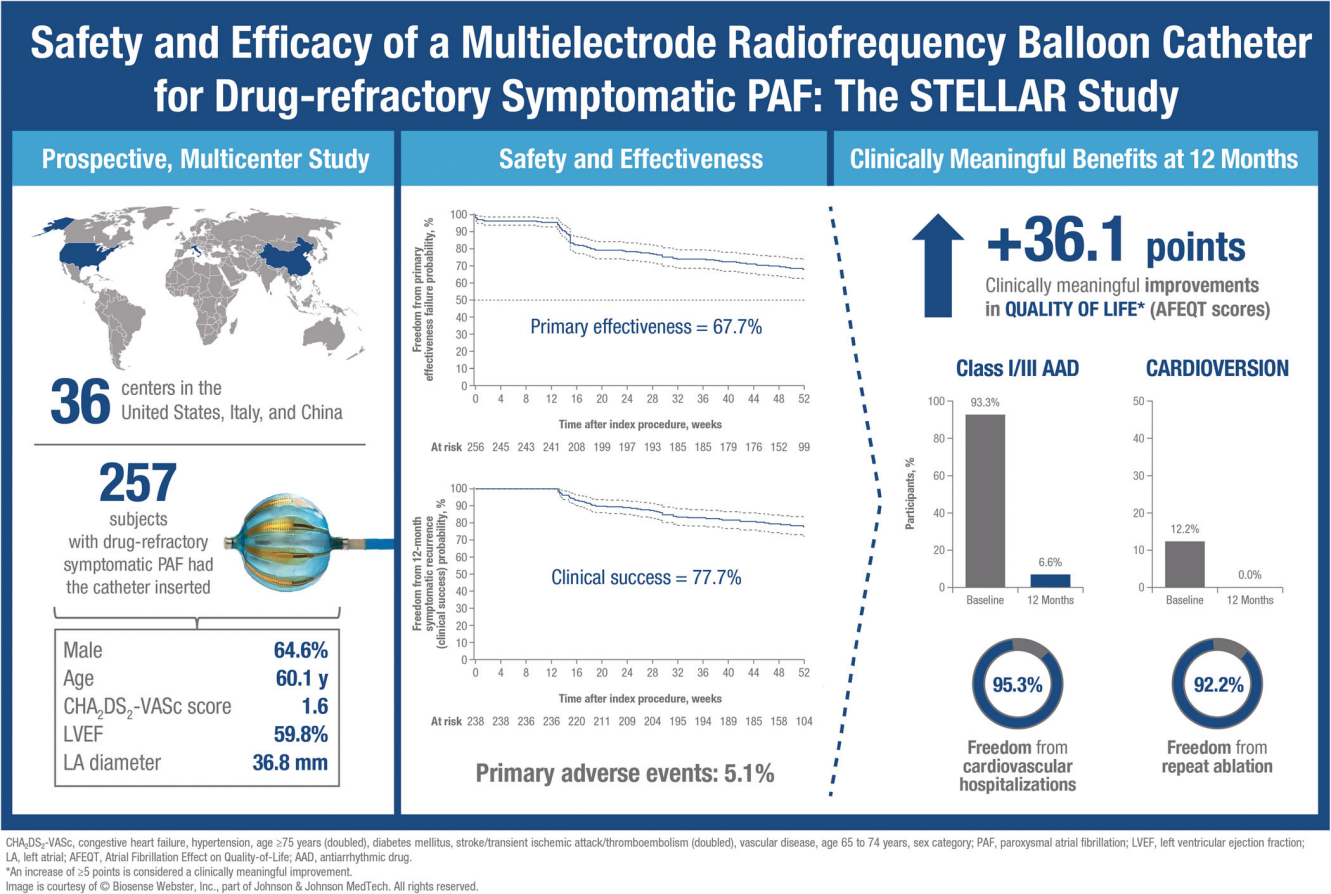
AAD, antiarrhythmic drug; AFEQT, atrial fibrillation effect on quality‐of‐life; CHA_2_DS_2_‐VASc, congestive heart failure, hypertension, age ≥75 years (doubled), diabetes mellitus, stroke/transient ischemic attack/thromboembolism (doubled), vascular disease, age 65–74 years, sex category; LA, left atrial; LVEF, left ventricular ejection fraction; PAF, paroxysmal atrial fibrillation. *An increase of ≥5 points is considered a clinically meaningful improvement. Image is courtesy of © Biosense Webster, Inc., part of Johnson & Johnson MedTech. All rights reserved.

## 2 Methods

### 2.1 Study Design and Participants

STELLAR was a prospective, multicenter, single‐arm clinical study conducted at sites in the United States, Italy, and China. The study was reviewed and approved by the institutional review board or ethics committee at all participating sites, and by all national authorities in the participating countries. All subjects provided written informed consent. Eligible participants were aged 18–75 years and had symptomatic PAF including the occurrence of ≥ 2 symptomatic atrial fibrillation (AF) episodes within the 6 months before enrollment and ≥ 1 AF episode documented via electrocardiogram (ECG) within 12 months before enrollment. Additionally, enrolled participants were required to have failed ≥ 1 Class I/III antiarrhythmic drug (AAD). Exclusion criteria included AF secondary to reversible or noncardiac causes, previous surgical or catheter ablation for AF, anticipated reception of ablation other than PVI, and persistent AF. Full inclusion and exclusion criteria are summarized in Supporting Information S1: Table S1.

### 2.2 Ablation Procedure for AF

Participants received anesthesia or sedation as per the individual site's standard practice. Appropriate strategies were employed to minimize the risk of esophageal injury by ensuring the operating physician was aware of the location of the esophagus relative to the intended ablation sites. At minimum, the use of an esophageal probe for temperature monitoring was required. Under the rare circumstance a temperature probe was not possible, ≥ 1 of the following alternative methods for esophageal protection was required: (1) esophageal visualization using barium swallow or contrast and/or (2) esophageal visualization with CARTOSOUND (Biosense Webster, Inc., Irvine, CA) and/or intracardiac echocardiography (ICE).

Following successful single transseptal puncture, mapping of the left atrium (LA) was performed. A 13.5 F sheath was used to insert the balloon catheter in the LA, and an activated clotting time of ≥ 350 s was confirmed before insertion and was maintained throughout the procedure. Before extending the balloon outside the sheath, the circular diagnostic catheter was advanced in the target vein under fluoroscopy and mapping guidance; correct positioning of the compliant multielectrode RF balloon catheter in the PV ostium was confirmed on the CARTO System (Biosense Webster, Inc., Irvine, CA), including evaluation of proximity to the phrenic nerve. To prevent phrenic nerve paralysis, the procedure workflow included requirements to pace the phrenic nerve when ablating the right‐sided veins and to conduct a fluoroscopic evaluation of the diaphragm after all ablations were completed. Ablation was applied until PVI was achieved and confirmed with the circular diagnostic catheter. The amount of power delivered to each electrode could be set independently with temperature‐controlled unipolar energy delivery set at a maximum of 15 W and maximum electrode temperature at 55°C. The RF delivery time was 20 s (maximum 30 s) on posterior electrode(s) and a maximum of 60 s on anterior electrodes. Isoproterenol/adenosine challenge was then administered for all targeted PVs to confirm entrance block; the study protocol did not mandate an additional waiting period. If PVI was not achieved, additional RF applications were applied using the HELIOSTAR catheter. If necessary, and at the investigator's discretion, a commercially available RF focal catheter was then used to complete PVI. Ablation outside the PV or in the cavotricuspid isthmus (CTI) region with a focal RF catheter was allowed only if arrhythmias were identified or triggered during the procedure.

### 2.3 Rhythm Monitoring

Arrhythmia monitoring during follow‐up included weekly transtelephonic monitoring (TTM) from 1 to 5 months and monthly from 6 to 12 months, all symptomatic cardiac episodes were recorded and transmitted via TTM at the time of event(s). In addition, a 24‐h Holter monitor was recorded at 12 months. Standard 12‐lead ECG recordings were collected at 1, 3, and 12‐month visits. All TTM tracings and Holter recordings were assessed by an independent cardiologist for adjudication.

### 2.4 Study Endpoints

The primary effectiveness endpoint was freedom from documented asymptomatic and symptomatic AF, atrial tachycardia (AT), or atrial flutter (AFL) episodes based on ECG data (≥ 30s on arrhythmia monitoring device) through the effectiveness evaluation period (days 91–365). Occurrence of any of the following was also considered a primary effectiveness failure: acute procedural failure (failure to confirm entrance block in all clinically relevant PVs); use of nonstudy catheter for isolation of clinically relevant PVs; > 2 repeat ablations during the blanking period (≤ 90 days postindex procedure) or any repeat ablation or surgical treatment for AF/AT/AFL during the evaluation period; direct current cardioversion (DCCV) during the evaluation period; recurrence of continuous AF/AT/AFL during the evaluation period; and Class I/III AADs prescribed/taken for atrial arrhythmia other than CTI‐dependent AFL at any time beyond the 3‐month follow‐up visit window (i.e., days 105–365 postindex procedure); or oral amiodarone prescribed after the initial ablation procedure. The primary effectiveness performance goal was 50%.

The primary safety endpoint was the occurrence of any primary adverse event (PAE) within 7 days following the initial ablation procedure, including myocardial infarction, stroke, thromboembolism, transient ischemic attack, permanent phrenic nerve paralysis, pulmonary edema, pericarditis, major vascular access complications, and hospitalization (initial or prolonged). Device‐ or procedure‐related death, atrioesophageal fistula, cardiac tamponade/perforation, and PV stenosis occurring later than 7 days after the ablation procedure were also considered PAEs. The primary safety performance goal was 14%.

Additional evaluations included acute procedural success (defined as confirmation of entrance block in clinically relevant PVs after adenosine/isoproterenol challenge), 12‐month freedom from asymptomatic or symptomatic AF/AT/AFL recurrence, and 12‐month freedom from symptomatic AF/AT/AFL recurrence (clinical success). The following procedural data were also collected: total duration of mapping, duration of RF application, balloon LA dwell time, fluoroscopy time, overall procedure time, first pass isolation (defined as PVI without acute reconnection before adenosine challenge), and number of repeat ablation procedures. To allow for an evaluation of the safety and effectiveness that is not diminished by the early learning, the first 1–3 subjects treated by an ablating physician with the compliant multielectrode RF balloon catheter were prospectively assigned as roll‐in subjects, with the number of required roll‐in cases per physician dependent on the physician's level of prior balloon catheter ablation experience.

### 2.5 Neurologic Assessment

In a subset of patients, incidences of silent cerebral lesions (SCLs) were evaluated using diffusion‐weighted magnetic resonance imaging (MRI) within the 72 h before the ablation procedure, as well as diffusion‐weighted and fluid‐attenuated inversion recovery (FLAIR) imaging within 12–48 h postprocedure. Patients with identifiable lesions or neurologic symptoms had a follow‐up MRI to determine lesion progress until lesions were resolved. All patients underwent neurologic and cognitive assessments, including National Institutes of Health Stroke Scale (NIHSS) assessments preablation and within 24–48 h postprocedure.

### 2.6 Assessment of PV Stenosis

All participants underwent a baseline cardiac computed tomography or magnetic resonance angiogram assessment (CT/MRA), while a subset also underwent a CT/MRA at 3 months after ablation to assess the incidence of postablation severe PV stenosis. In addition to this cardiac CT/MRA subset, any participants who developed signs or symptoms of PV stenosis underwent a postablation CT/MRA. These subjects were not analyzed in the CT/MRA subset. If severe PV stenosis was present, it was reported as an adverse event.

### 2.7 Quality of Life Assessment and Clinical Benefits

The Atrial Fibrillation Effect on Quality‐of‐Life (AFEQT) questionnaire is an AF‐specific health‐related quality‐of‐life questionnaire that includes 20 questions grouped into 4 functional subscales: symptoms, daily activities, treatment concern, and treatment satisfaction. The AFEQT scores were assessed preablation and at 3, 6, and 12 months postablation. Additionally, the following clinical outcomes were assessed after the ablation procedure: 12‐month freedom from cardiovascular hospitalization, incidence of DCCV procedures postablation, and use of Class I/III AADs.

### 2.8 Statistical Analysis

The safety analysis set was composed of all enrolled participants who underwent insertion of the study catheter. The modified intent‐to‐treat (mITT) analysis set consisted of enrolled participants who met eligibility criteria and had the study catheter inserted. The per‐protocol (PP) analysis set, a subset of the mITT population, was treated for study‐related arrhythmia without major protocol deviation. The neurologic assessment evaluable (NAE) subset was composed of the first participants consecutively enrolled in the study at participating NAE sites who signed the NAE consent form and met study and NAE eligibility criteria and completed SCL assessment preablation and postablation MRIs. The cardiac CT/MRA subset for PV stenosis assessment was composed of the first 40 participants consecutively enrolled in the study at participating cardiac CT/MRA sites who have readable outcomes at baseline and 3 months. The primary safety and effectiveness endpoints were analyzed for the mITT analysis set, while analyses of all secondary endpoints were performed descriptively in the PP analysis set.

A Wilcoxon rank‐sum test for continuous data was used to compare differences in procedural data and treatment outcomes between patients receiving ablation under general anesthesia versus conscious sedation. Procedure time, fluoroscopy time, and LA balloon dwell time were summarized by mean and standard deviation for the roll‐in cases and main study cases.

The impact of operators’ learning curves on workflow factors was calculated per operator using the roll‐in and PP analysis sets. For each center, procedural data from the first roll‐in case to the last PP case were plotted against the number of ablations. Learning curves were analyzed for total procedural and balloon dwell times, as well as fluid delivery via the study catheter.

## 3 Results

### 3.1 Study Population

**Figure 1:**
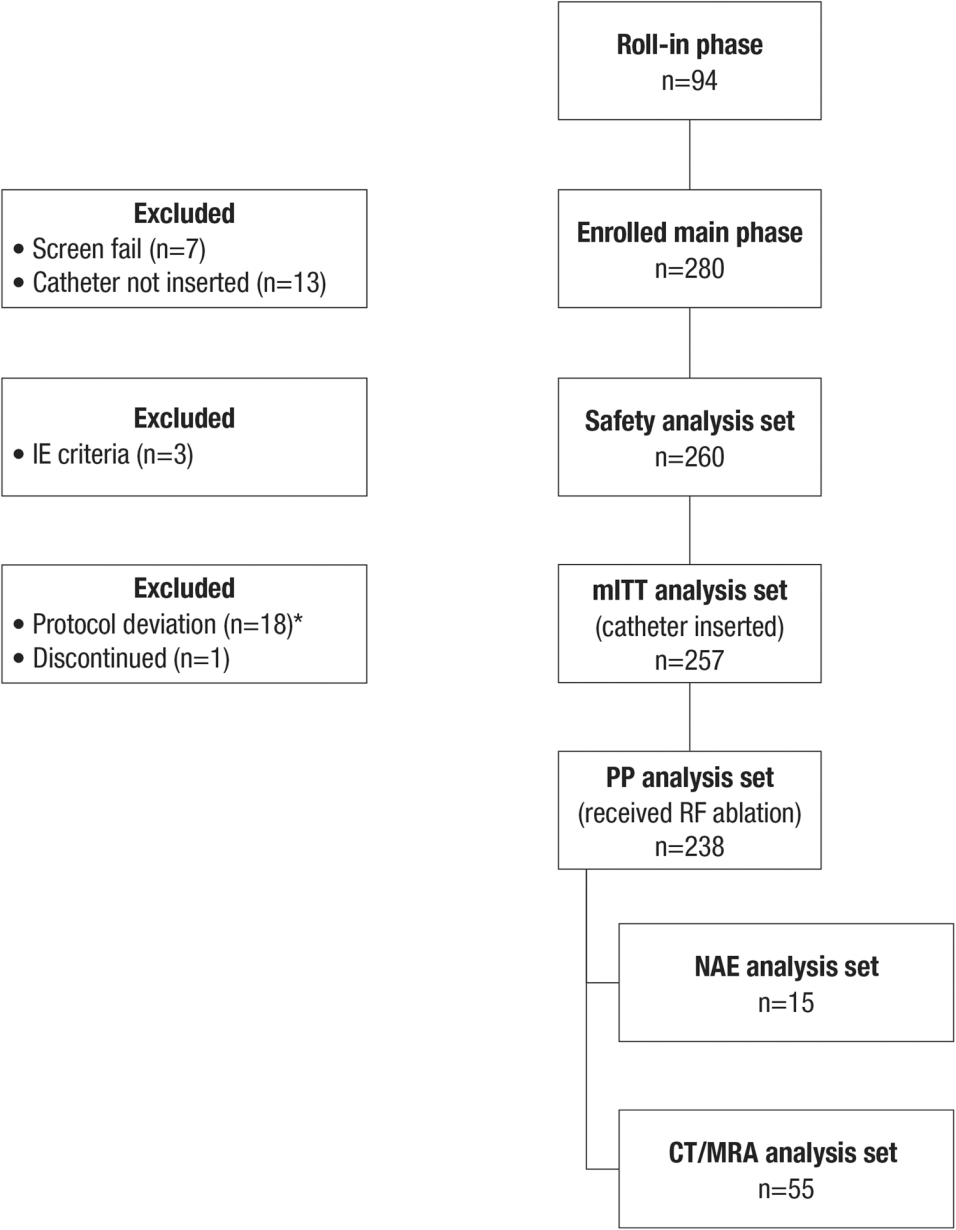
Participant disposition. CT, computed tomography; IE, inclusion and exclusion; mITT, modified intent‐to‐treat; MRA, magnetic resonance angiogram; NAE, neurological assessment evaluable; PP, per‐protocol. *Details of protocol deviations are provided in Supporting Information S1: Table S3.

A total of 36 study sites enrolled participants (United States, 32; Italy, 2; China, 2; Supporting Information S1: Table S2). Of 280 participants enrolled in the main phase, 257 met inclusion criteria for the safety and effectiveness evaluation (mITT; Figure 1). Baseline characteristics and medical history of the participants in the safety population are shown in Table 1. Most of the patients were White (81.9%) and male (64.6%). Mean age was 60.1 years, mean CHA_2_DS_2_‐VASc score was 1.6, and mean LA diameter was 36.8 mm. The most common baseline comorbidities were hypertension (55.4%) and obstructive sleep apnea (21.2%). The PP analysis was performed on 238 patients, while 22 patients in the safety analysis set were excluded due to protocol deviation (shown in Supporting Information S1: Table S3).

**Table 1:** Baseline characteristics and medical history (safety analysis set, *n* = 260).

	Safety analysis set, *n* = 260
Age (years), mean (SD)	60.1 (9.19)
Male, *n* (%)	168 (64.6)
Race, *n* (%)	
Asian	21 (8.1)
Black/African American	7 (2.7)
White	213 (81.9)
Not reported	19 (7.3)
Time since start of symptomatic AF (months), mean (SD)	52.1 (67.8)
CHA_2_DS_2_‐VASc score, mean (SD)	1.6 (1.3)
Left atrial diameter (mm), mean (SD)	36.8 (6.2)
Left ventricle ejection fraction (%), mean (SD)	59.8 (6.1)
Pharmacologic cardioversion in the past 12 months, *n* (%)	39 (15.0)
DCCV in the past 12 months, *n* (%)	31 (11.9)
Number of AADs failed, mean (SD)	1.3 (0.6)
Class I/III AADs, *n* (%)*	241 (92.7)
Amiodarone*	11 (4.6)
Comorbidities, *n* (%)	
Coronary artery disease	34 (13.1)
Congestive heart failure, NYHA Class I	2 (0.8)
Hypertension	144 (55.4)
Type 2 diabetes mellitus	31 (11.9)
Thromboembolic events	4 (1.5)
Atrial flutter	50 (19.2)
Obstructive sleep apnea	55 (21.2)

*Note:* *Percent calculated based on subjects with available data.

Abbreviations: AAD, antiarrhythmic drug; AF, atrial fibrillation; CHA_2_DS_2_‐VASc, congestive heart failure, hypertension, age ≥75 years (doubled), diabetes mellitus, stroke/transient ischemic attack/thromboembolism (doubled), vascular disease, age 65–74 years, sex category; DCCV, direct current cardioversion; NYHA, New York Heart Association; SD, standard deviation.

### 3.2 Procedural Characteristics

**Figure 2:**
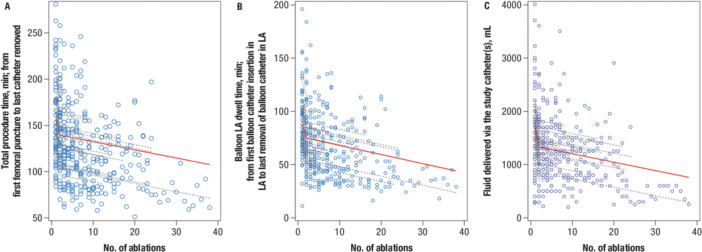
Operator learning curve for (A) total procedure time, (B) LA balloon dwell time, and (C) fluid delivery via study catheter. LA, left atrium.

Procedural characteristics are summarized in Table 2. In the PP analysis set, the mean total procedure time was 116.4 ± 37.0 min, and the mean total fluoroscopy time during balloon phase was 13.5 ± 9.3 min. Total procedure time is defined as the time of first venous puncture until the time of last catheter removal, thus including time needed for transseptal puncture, mapping, ablation, and challenge with adenosine/isoproterenol. The mean balloon atrial dwell time, defined as the time from balloon insertion in the LA until the balloon was removed from the LA, was 60.2 ± 26.5 min. After the first procedure among all operators, total procedure time decreased by 16.0 min, balloon left atrial dwell time decreased by 12.1 min, and fluid delivery via the study catheter decreased by 331.1 mL (Figure 2).

**Table 2:** Ablation procedural characteristics (PP analysis set, *n* = 238).

	PP analysis set, *n* = 238
General anesthesia, *n* (%)	191 (80.3)
Total procedure time (min)	116.4 (37.0)
Balloon dwell time (min)	60.2 (26.5)
Fluoroscopy time during balloon phase (min)	13.5 (9.3)
Total mapping time (min)	11.0 (16.9)
Total duration of RF applications (min)	8.3 (3.3)
Total number of valid RF applications per vein	
LSPV, *n* = 230	3.0 (2.3)
LIPV, *n* = 230	2.3 (1.7)
RSPV, *n* = 237	2.3 (1.7)
RMPV, *n* = 13	1.0 (0.0)
RIPV, *n* = 236	2.1 (1.5)
LVP (common), *n* = 8	3.9 (2.6)
Fluid delivered via study catheter (mL)	1147.8 (562.9)
PVI with entrance block confirmed in targeted PVs, *n* (%)	238 (100)
PVI with only balloon catheter, *n* (%)	224 (94.1)

*Note:* Reporting mean (SD) unless stated otherwise.

Abbreviations: LIPV, left inferior pulmonary vein; LSPV, left superior pulmonary vein; LVP, left pulmonary vein; PP, per‐protocol; PV, pulmonary vein; PVI, pulmonary vein isolation; RF, radiofrequency; RIPV, right inferior pulmonary vein; RMPV, right middle pulmonary vein; RSPV, right superior pulmonary vein; SD, standard deviation.

Acute procedural success, defined as entrance block confirmed in targeted PVs, was achieved for all 238 participants in the PP analysis set (100%). In 94.1% (224/238) cases acute procedural success was achieved with the balloon catheters alone but 5.9% patients required a separate ablation catheter to complete PVI at the investigator's discretion. The first pass isolation rate was 87.4% (834/954) at the vein level. The cases requiring use of a separate ablation catheter to achieve PVI were considered as primary effectiveness failures. Ablation procedures performed under conscious sedation generally led to shorter procedure time, mapping time, and LA dwell time compared with general anesthesia (Supporting Information S1: Table S4).

### 3.3 Follow‐Up Compliance

For the mITT analysis set, the weekly TTM compliance rates ranged from 60.3% to 72.2%, and the monthly compliance rates beginning at month 6 ranged from 70.2% to 80.7%. The ECG compliance rate was 97.2% at month 3, 92.8% at month 6, and 94.8% at month 12. Holter monitor compliance rate was 93.5% at the 12‐month visit.

### 3.4 Effectiveness and Safety

**Figure 3:**
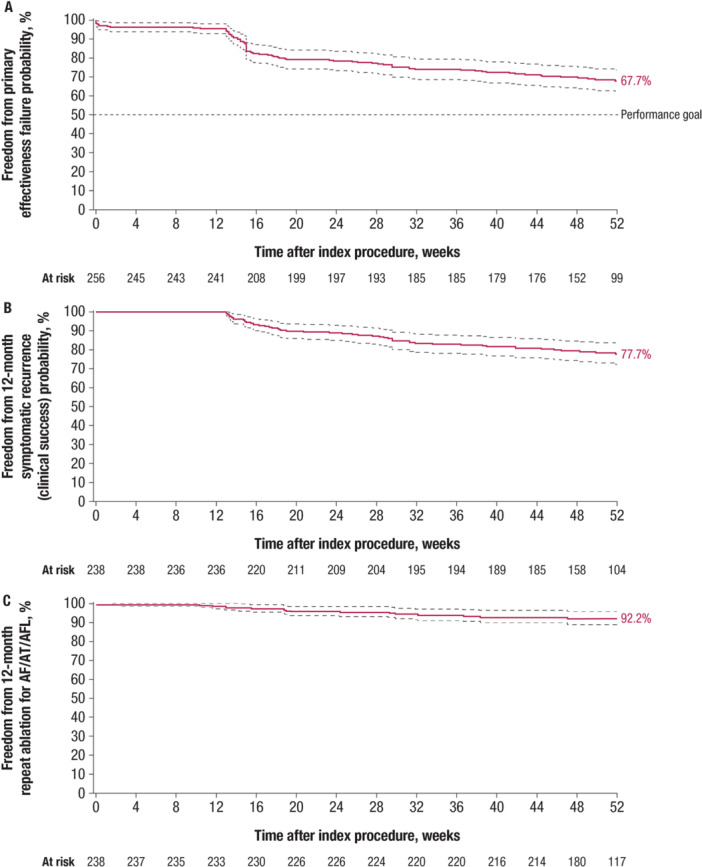
Kaplan–Meier analysis of (A) time to primary effectiveness failure (mITT analysis set, *n* = 257), (B) time to symptomatic recurrence (clinical success; PP analysis set, *n* = 238), and (C) time to repeat ablation for AF/AT/AFL (PP analysis set, *n* = 238). AF, atrial fibrillation; AFL, atrial flutter; AT, atrial tachycardia; mITT, modified intent‐to‐treat; PP, per‐protocol.

The Kaplan–Meier estimate of 12‐month primary effectiveness was 67.7% (95% LCB, 61.9%), thereby meeting the performance goal of 50% (Figure 3A). One‐year clinical success (freedom from documented symptomatic AF/AT/AFL recurrence) was 77.7% (Figure 3B), 12‐month freedom from all documented symptomatic or asymptomatic recurrence was 75.1%, and 12‐month freedom from repeat ablation was 92.2% (Figure 3C).

For the primary safety endpoint, there were 13 PAEs reported in 13 participants (raw PAE rate: 5.1%; posterior mean: 5.45%; 95% BCI: 3.02%–8.53%; Table 3), which met the 14% performance goal.

**Table 3:** PAEs (mITT analysis set, *n* = 257).

PAEs	No. of participants with event	No. of events	Event rate, *n* (%)^a^
Any PAE	13	13	13 (5.1)
Device‐ or procedure‐related death	1	1	1 (0.4)
Severe pulmonary vein stenosis	1	1	1 (0.4)
Atrioesophageal fistula	0	0	0 (0)
Myocardial infarction	0	0	0 (0)
Cardiac tamponade/perforation	0	0	0 (0)
Thromboembolism	0	0	0 (0)
Stroke/cerebrovascular accident	1	1	1 (0.4)
Transient ischemic attack	1	1	1 (0.4)
Phrenic nerve paralysis (permanent)	2	2	2 (0.8)
Pericarditis	1	1	1 (0.4)
Pulmonary edema	0	0	0 (0%)
Major vascular access complication/bleeding	2	2	2 (0.8)
Hospitalization (initial or prolonged)^b^	4	4	4 (1.6)

Abbreviations: mITT, modified intent‐to‐treat; PAE, primary adverse event.

a Event rate is the percentage of participants with the event. Only participants with nonmissing data (*n* = 255) were included.

^b^ Excludes hospitalization solely due to arrhythmia recurrence or nonmedically urgent cardioversion.

There were no reports of atrioesophageal fistula, myocardial infarction, cardiac tamponade or perforation, thromboembolism, or pulmonary edema. Following appropriate management, nine PAEs were considered resolved or recovered; one (a minor stroke/cerebrovascular accident) was reported as recovered with sequelae, two (a phrenic nerve injury and a PV stenosis) were reported as not recovered or resolved, and one (a PV‐bronchial fistula which was initially diagnosed as acute bronchitis) resulted in the patient's death 42 days after ablation. Analysis of the subset of cardiac CT/MRA cohort showed 29 participants (52.7%) with mild asymptomatic PV stenosis (> 20%–50% reduction in PV diameter) requiring no treatment. There were no instances of moderate or severe PV stenosis in the CT/MRA analysis set (*n* = 55). One subject not in the CT/MRA subset experienced PV stenosis and this was reported as a PAE.

### 3.5 Assessment of SCL

One of the 15 participants (6.7%) in the NAE subset had an asymptomatic SCL at 1 month postprocedure; it resolved at the 3‐month follow‐up with no clinical manifestation.

### 3.6 Quality of Life and Clinical Benefits

**Figure 4:**
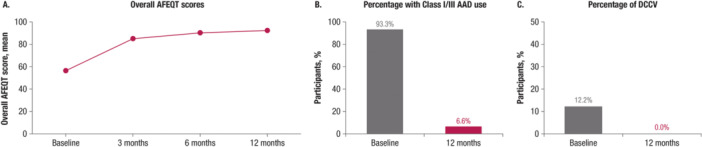
Quality of life and clinical benefits (A) overall AFEQT scores (PP analysis set, *n* = 238), (B) percentage of patients receiving Class I/III AAD (PP analysis set, *n* = 238), (C) percentage of patients who had DCCV scores (PP analysis set, *n* = 238). AAD, antiarrhythmic drug; AFEQT, atrial fibrillation effect on quality‐of‐life; DCCV, direct current cardioversion; PP, per‐protocol.

The mean improvements in AFEQT scores at 3, 6, and 12 months after ablation indicated a sustained and clinically meaningful improvement in health‐related quality of life (>5 points [11]) over time of +36.1 points (Figure 4A); the Kaplan–Meier estimate of freedom from cardiovascular hospitalizations at 12 months postprocedure was 95.3%. The percentage of subjects receiving Class I/III AADs decreased from 93.3% (222/238) at baseline to 6.6% (15/229) at 12 months (Figure 4B), while the percentage of subjects who have had DCCV decreased from 12.2% (29/238) at baseline to 0.0% (0/229) at 12 months (Figure 4C).

## 4 Discussion

The results of the STELLAR study demonstrated the effectiveness, safety, and clinical benefits of the HELIOSTAR compliant multielectrode RF balloon catheter in PAF ablation. Primary effectiveness and clinical success were 67.7% and 77.7%, respectively, with a PAE rate (5.1%) consistent with prior results of new balloon devices [12, 13]. In addition, there was clinically meaningful improvement in patients’ quality of life. Substantial reductions in healthcare utilization including AAD use (–86.7%) and cardioversion (–12.2%) reductions, as well as high rates of freedom from repeat ablation (> 90%) and cardiovascular hospitalization (> 95%) postablation were also observed.

The rate of freedom from atrial arrhythmia recurrence observed in the current study (75.1%) compares favorably with earlier clinical experience from the SHINE study (65.8%) [7], and is similar to those reported in recent studies of PBP RF catheter or cryoballoon ablation. In the European VISTAX and the US SURPOINT postapproval studies of PBP RF catheter ablation using a tag index under a standardized PVI workflow, the rates of freedom from any documented atrial arrhythmia were 78.3% and 81.5%, respectively, at 12‐month follow‐up using stringent monitoring [1, 2]. Similarly, the Q‐FFICIENCY study of high‐power, short‐duration, temperature‐controlled RF catheter ablation of PAF demonstrated a 12‐month freedom from atrial tachyarrhythmia recurrence rate of 82.1% [14]. The CIRCA‐DOSE trial of 2‐ or 4‐min cryoballoon ablation compared with RF catheter ablation showed an overall freedom from atrial arrythmia of 53% with continuous arrhythmia recording [15]. However, this translated to a 12‐month success rate of approximately 76% when using monitoring similar to this study [16]. The FIRE and ICE study reported a 12‐month success of 64%–65% for both PBP RF ablation and cryoballoon ablation groups [17].

This study met the primary safety endpoint. There were no reports of atrioesophageal fistula, myocardial infarction, cardiac tamponade or perforation, thromboembolism, or pulmonary edema. The rate of PAEs reported (5.1%) was comparable to previously published ranges of RF catheter ablation, both with PBP and balloon catheters [1, 2, 18]. Only 1 participant experienced an SCL, which was resolved at the 3‐month follow up. Clinical occurrence of SCL is a known risk of catheter ablation with heretofore unknown clinical significance. Although SCL rates in published literature range widely and are as high as 38% [19], much has been learned from prior studies in terms of workflow adjustment to minimize their occurrence [20, 21]. Employing an improved workflow based on these prior experiences, the current study results showed a risk of SCL with the compliant multielectrode RF balloon catheter comparable to other recent ablation technologies [22, 23]. Although 52.7% of scanned participants had mild PV narrowing, none of them had clinical symptoms or required any treatments [24]. Among the series of AF ablation reports reviewed for the 2017 HRS/EHRA/ECAS consensus document on balloon‐based AF ablation, permanent phrenic nerve paralysis was reported as a known complication with an incidence of up to 1.8% at 1 year [24, 25]. In our study, 2 out of 255 subjects (0.8%) had phrenic nerve injury/diaphragmatic paralysis. After these two phrenic nerve paralyses were observed, additional strategies were included in the procedure workflow that: (1) ensured that paralytics were either not used or their effects reversed before attempting to ablate the right sided veins, (2) recommended pacing from the anterior balloon electrodes to identify the balloon's proximity to the phrenic nerve, and (3) required operators to document the integrity of the phrenic nerve at the end of the case through fluoroscopic demonstration of diaphragmatic contraction while pacing. After the implementation of these mitigations, no documented phrenic nerve injury occurred. This finding showcases the effects of learning curve and reiterates the importance of workflow. In addition, one subject experienced a left PV left bronchial fistula with an outcome of death. Bronchial injury has been reported in focal RF or cryoballoon ablations [26, 27]. In this patient, unusual workflow characteristics were used delivering a higher amount of RF, and additional ablations were performed near LSPV, even after the LSPV was isolated. Another possible reason is that the balloon catheter might ablate the more distal part of the PV (near the bronchus) rather than the proximal part, playing a role in the occurrence of the bronchial injury. This finding underscores the need to limit RF delivery to the posterior wall to recommended parameters, even if the esophagus is deemed to be away from the area of ablation. This event represented a device‐ or procedure‐related death rate of 0.4% (1/255) in the mITT analysis set. This rate falls within the incidence rate range (up to 1.5%) reported in the 2017 HRS/EHRA/ECAS Expert Consensus Statement [24, 28].

Total procedural time in the current study (116 min) was relatively longer than in the smaller SHINE study (88 min) [7]; this may be due to a lower use of general anesthesia in the SHINE trial (54%). This is consistent with observation from our study of the procedural data differences in the general anesthesia versus conscious sedation groups (Table 2 and Supporting Information S1: Table S4), as well as by European centers’ experience, [29] where procedures performed under conscious sedation are associated with higher procedural efficiency compared with general anesthesia, without significant impact on safety or efficacy. Total procedural time was shorter than that reported in recent multicenter studies using focal RF catheters (132–156 min) [1, 2, 14] or the cryoballoon (131–143 min) [15]. Short learning curves were reported in this study, with reductions in total procedure and balloon LA dwell times demonstrated for all operators after their initial procedure, indicating an improvement in procedural efficiency.

Clinically impactful benefits to patients who underwent ablation procedures were seen in improvements in AF‐related quality of life. The overall AFEQT score showed a significant improvement at 3 months compared with baseline (+29 points), which was sustained up to 12 months after the procedure (+36 points). These improvements in the AFEQT score exceeded the minimum clinically important difference in an individual patient by >5 points [11]. Healthcare utilization reduction was also observed, with >90% to 95% of patients free from cardiovascular hospitalization and repeat ablations, as well as significant reduction in rates of direct‐current cardioversion and Class I/III AAD use after ablation. These improvements are clinically meaningful and may translate to long‐term savings in the cost of care for these patients.

### 4.1 Study Limitations

Limitations of the current study include its nonrandomized single‐arm design, as well as the lack of a waiting period after the ablation. A randomized controlled study would be required to draw direct comparisons of the clinical safety and efficacy with other AF ablation technologies.

## 5 Conclusion

The STELLAR study demonstrated the safe and effective use of the new compliant multielectrode RF balloon catheter for the treatment of symptomatic drug‐refractory PAF, with clinically meaningful improvements in quality of life and reductions in healthcare utilization.

## 6 Competency in Medical Knowledge

In a multinational clinical evaluation, ablation with the HELIOSTAR RF balloon catheter provided freedom from symptomatic atrial tachyarrhythmias of 78%, a low PAE rate of 5.1%, and clinically meaningful improvements in AF‐related clinical benefits and quality of life.

## 7 Translational Outlook

Further studies are needed to support the reproducibility of the RF balloon catheter ablation long‐term safety and effectiveness, but the catheter is no longer commercially available.

## Ethics Statement

This study was reviewed and approved by the institutional review board or ethics committee at all participating sites, and by all national authorities in the participating countries.

## Consent

All subjects provided written informed consent.

## Conflicts of Interest

S.K.G. has received consulting fees and/or honoraria from Biosense Webster, Medtronic, and Galaxy Medical. C.P. has received consulting fees and/or honoraria from Biosense Webster. M.G. has received honoraria for lectures and presentations from Biosense Webster and has patent agreements with Biosense Webster. S.M. has received grants from Abbott, Medtronic, and Biotronik; consulting fees from Biosense Webster; honoraria from Boston Scientific, Zoll, and Biotronik; and support for attending meetings and/or travel from Medtronic and Biotronik. J.B.D. has received consulting fees and grants from Biosense Webster. C.‐Y.J. has received grants and payment or honoraria for lectures and presentations from Biosense Webster and payment or honoraria for lectures and presentations from Boston Scientific and Abbott. S.D. has received royalty payments from Boston Scientific and owns stock or stock options in Manual Surgical Sciences, LLC. H.C. has received consulting fees and/or honoraria from Biosense Webster, Medtronic, Boston Scientific, Abbott, and AtriCure. M.M. has received consulting fees and/or honoraria from Biosense Webster, Medtronic, Boston Scientific, and Siemens; and owns stock or stock options in NewPace Ltd and EPD Solutions. A.N. has received consulting fees from Biosense Webster, Abbott, Boston Scientific, Biotronik, Baylis, and Medtronic. G.C. has received consulting fees from Biotronik. A.A. has received consulting fees and grants from Biosense Webster. S.W.L., V.S.S., H.J., A.P.W., L.‐Q.W., H.R., and J.R.‐G. declare no conflicts of interest.

## Supporting information

Supporting information.

## Data Availability

The data that support the findings of this study are available from the corresponding author upon reasonable request. Johnson & Johnson MedTech has an agreement with the Yale Open Data Access (YODA) Project to serve as the independent review panel for the evaluation of requests for clinical study reports and patient‐level data from investigators and physicians for scientific research that will advance medical knowledge and public health. Requests for access to the study data can be submitted through the YODA Project site at http://yoda.yale.edu.
