# Supplementary material for: Multielectrode Radiofrequency Balloon Catheter for Paroxysmal Atrial Fibrillation: Results From the Global, Multicenter, STELLAR Study
Source: J Cardiovasc Electrophysiol. 2024 Dec 16;36(2):376–86. doi: 10.1111/jce.16524 (PMC11837878; doi:10.1111/jce.16524)
Supplement: Supplementary file 1 — Supporting information. [file JCE-36-376-s001.docx]

**Supplementary Materials**

**Supplementary Table 1. Patient Inclusion and Exclusion Criteria.**

| **Inclusion criteria** | - Diagnosed with symptomatic PAF (physician’s note indicating recurrent self-terminating AF)   - ≥2 symptomatic AF episodes within the last 6 months from enrollment   - ≥1 electrocardiographically documented AF episode within 12 months prior to enrollment. electrocardiographic documentation may include, but is not limited to, ECG, Holter monitor, or telemetry strip - Failed ≥1 AAD (Class I/III) as evidenced by recurrent symptomatic AF, contraindication to the AAD, or intolerable adverse effects to the AAD - Age 18 to 75 years - Able and willing to comply with all pretesting, posttesting, and follow-up testing and visit requirements - Signed patient informed consent form |
| --- | --- |
| **Exclusion criteria** | - AF secondary to electrolyte imbalance, thyroid disease, or reversible or noncardiac cause (eg, documented obstructive sleep apnea, acute alcohol toxicity) - Previous surgical or catheter ablation for AF - Patients known to require ablation outside the PV ostia and CTI region (eg, atrioventricular re-entrant tachycardia, atrioventricular nodal re-entry tachycardia, AT, ventricular tachycardia, Wolff-Parkinson-White) - Previously diagnosed with persistent or long-standing persistent AF and/or continuous AF lasting >7 days - Any percutaneous coronary intervention within the past 2 months - Valve repair or replacement or presence of a prosthetic valve - Any carotid stenting or endarterectomy within the past 6 months - Coronary artery bypass grafting, cardiac surgery (eg, ventriculotomy, atriotomy), or valvular cardiac surgical procedure within the past 6 months - Documented LA thrombus within 1 day prior to the index procedure - LA anteroposterior diameter >50 mm - LVEF <40% - Contraindication to anticoagulation (eg, heparin) - History of blood clotting or bleeding abnormalities - Myocardial infarction within the past 2 months - Documented thromboembolic event (including transient ischemic attack) within the past 12 months - Rheumatic heart disease - Uncontrolled heart failure or NYHA functional class III/IV - Awaiting cardiac transplantation or other cardiac surgery within the next 12 months - Unstable angina - Acute illness or active systemic infection or sepsis - Diagnosed atrial myxoma or presence of an interatrial baffle or patch - Presence of an implanted pacemaker or ICD - Significant pulmonary disease (eg, restrictive pulmonary disease, constrictive or chronic obstructive pulmonary disease) or any other disease or malfunction of the lungs or respiratory system that produces chronic symptoms - Significant congenital anomaly or medical problem that, in the opinion of the investigator, would preclude enrollment in this study - Women who are pregnant (as evidenced by pregnancy test if premenopausal), lactating, or who are of child-bearing age and plan on becoming pregnant during the course of the clinical investigation - Enrolled in an investigational study evaluating another device, biologic, or drug - Has known pulmonary vein stenosis - Presence of intramural thrombus, tumor, or other abnormality that precludes vascular access or manipulation of the catheter - Presence of an inferior vena cava filter - Presence of a condition that precludes vascular access - Life expectancy or other disease processes likely to limit survival to <12 months - Presenting contraindication for the devices (eg, TTE, Holter, CT) used in the study, as indicated in the respective instructions for use |
| **Additional exclusion criteria for the NAE subset** | - Contraindication to use of contrast agents for MRI, such as advanced renal disease, et cetera - Presence of iron-containing metal fragments in the body - Unresolved pre-existing neurologic deficit |

AAD, antiarrhythmic drug; AF, atrial fibrillation; AT, atrial tachycardia; CT, computed tomography; CTI, cavotricuspid isthmus; ECG, electrocardiogram; ICD, implantable cardioverter defibrillator; LA, left atrial; LVEF, left ventricular ejection fraction; MRI, magnetic resonance imaging; NAE, neurological assessment evaluable; NYHA, New York Heart Association; PAF, paroxysmal atrial fibrillation; PV, pulmonary vein; TTE, transthoracic echocardiogram.

**Supplementary Table 2. Study Sites and Principal Investigators**

| **Study Site** | **Principal Investigators** |
| --- | --- |
| AHS Hospital Corp  Morristown Medical Center | Timothy Mahoney, MD, MS |
| Baptist Heart Specialists | Venkata S. Sagi, MD, MBBS |
| CHI Baylor St Lukes  Hail Garcia Cardiology | Abdi Rasekh, MD |
| Doylestown Health Cardiology | Robert M. Sangrigoli, MD |
| Emory Saint Joseph’s Hospital | Anshul Patel, MD, FACC, FHRS |
| The Heart Hospital Baylor Plano | James Brian DeVille, MD, FACC, FHRS |
| Hoag Memorial Hospital | Michael S. Panutich, MD |
| Icahn School of Medicine at Mount Sinai/Cardiovascular Institute | Srinivas Dukkipati, MD |
| Inova Fairfax Medical Campus—Inova Heart and Vascular Institute | Haroon Rashid, MD |
| Johns Hopkins Hospital | Hugh Calkins, MD |
| Kansas City Cardiac Arrhythmia Research Institute  Overland Park Regional Medical Center | Dhanunjaya Lakkireddy, MD |
| Kettering Medical Center | Haseeb Jafri, MD |
| Lenox Hill Hospital | Stavros Mountantonakis, MD, MBA |
| Massachusetts General Hospital | Moussa Mansour, MD |
| Mayo Clinic Hospital | Douglas L. Packer, MD |
| Medical Center of the Rockies | Ethan Ellis, MD |
| Medstar Washington Hospital Center | Sung W. Lee, MD |
| Mercy General Hospital | Arash Aryana, MD, PhD, FACC, FHRS |
| Methodist Texan Hospital South Texas Cardiovascular | Javier Roman-Gonzalez, MD |
| Northwestern Memorial Hospital | Bradley P. Knight, MD, FACC, FHRS |
| Oklahoma Heart Institute | Craig S. Cameron, MD, FACC, FHRS |
| Phoenix Cardiovascular Research Group | Marwan M. Bahu, MD |
| Piedmont Heart Institute | Sandeep Kumar Goyal, MD |
| Saint Luke’s Cardiovascular Consultants | Alan P. Wimmer, MD, FACC, FHRS |
| Spectrum Health System | Sanjay Dandamudi, MD |
| Swedish Medical Center | Darryl S. Wells, MD, MBA, FACC, FHRS |
| Texas Cardiac Arrhythmia Research Foundation | Andrea Natale, MD, FHRS, FACC, FESC |
| The University of Kansas Health System | Yeruva Madhu Reddy, MD |
| UPMC Presbyterian | Andrew Howard Voigt, MD |
| The Valley Hospital | Suneet Mittal, MD |
| Vanderbilt University Medical Center | Sharon T. Shen, MD |
| Yale New Haven Hospital | James V. Freeman, MD, MPH, MS |
| Ruijin Hospital Shanghai Jiao Tong University School of Medicine—China | Li-Qun Wu, MD |
| Sir Run Run Shaw Hospital Zhejiang University School of Medicine—China | Chen-yang Jiang, MD, PhD |
| Ospedale Generale Regionale F. Miulli  UOC Cardiologia—Arrhythmias Unit—Italy | Massimo Grimaldi, MD |
| Policlinico Di San Donato Milanese  Arrhythmology Department—Italy | Carlo Pappone, MD, PhD; Giuseppe Ciconte MD |

**Supplementary Table 3. Major Prespecified Protocol Deviations for Removal From PP Analysis Set (Safety Analysis Set, n=260)**

| **Major protocol deviations resulting in exclusion from PP analysis set** | **No. of safety analysis set subjects** |
| --- | --- |
| Subjects were found not meeting eligibility criteria after undergoing the index procedure | 3 |
| All protocol specified electronic effectiveness* monitoring was missing | 2 |
| Failure to use the HELIOSTAR catheter for isolation of clinically relevant PVs (all PVs except those that were silent and/or cannot be cannulated) for repeat procedure(s) in blanking period | 2 |
| Class I/III AAD taken/prescribed at any time beyond the 3-month follow-up visit window (ie, at any time from day 105 to 365 post–index procedure) and/or oral amiodarone taken/prescribed postprocedure without documentation of arrhythmia† | 6 |
| Failure to check for entrance block for each targeted PV† | 3 |
| Failure to pace the phrenic nerve when ablating right sided veins | 6 |
| Study mandated catheter not used during procedure (PV isolation may be confirmed with alternative BWI diagnostic catheters as appropriate, instead of LASSOSTAR if needed) | 1 |

AAD, antiarrhythmic drug; BWI, Biosense Webster, Inc.; PP, per-protocol; PV, pulmonary vein.

*Electronic effectiveness monitoring included electrocardiograms, transtelephonic monitoring, and 24-hour Holter monitors. †One patient had both marked deviations.

**Supplementary Table 4. Procedural Data in Subjects Treated Under General Anesthesia Versus Conscious Sedation (PP Analysis Set, n=238)**

|  | **General anesthesia**  **(n=191)** | **Conscious sedation**  **(n=47)** | ***P* value** |
| --- | --- | --- | --- |
| Total procedure time (min), mean (SD) | 124.1 (35.92) | 85.2 (21.88) | <0.001 |
| Total mapping time (min), mean (SD) | 12.4 (18.6) | 5.7 (2.8) | <0.001 |
| Balloon dwell time (min), mean (SD) | 65.2 (26.5) | 39.5 (13.2) | <0.001 |

PP, per-protocol; SD, standard deviation.
